# Factors associated with an excellent transfer of passive immunity: multisite, cross-sectional study conducted in different European countries on dairy cattle

**DOI:** 10.3389/fvets.2025.1515196

**Published:** 2025-02-25

**Authors:** Aitor Fernandez-Novo, Iris Kolkman, Monique Driesse, Matt Yarnall, Manuel Cerviño, Francisco Javier Dieguez, Susana Astiz

**Affiliations:** ^1^Department of Veterinary Medicine, School of Biomedical and Health Sciences, Universidad Europea de Madrid, Villaviciosa de Odón, Spain; ^2^A7 Noord Dierenartsen, Drachten, Netherlands; ^3^Boehringer Ingelheim Animal Health Netherlands, Amsterdam, Netherlands; ^4^Boehringer Ingelheim Vetmedica GmbH, Ingelheim, Germany; ^5^Boehringer Ingelheim Animal Healh España, Sant Cugat del Vallés, Spain; ^6^Department of Anatomy, Animal Production and Clinical Veterinary Sciences, Veterinary Faculty, Santiago de Compostela University, Lugo, Spain; ^7^Animal Reproduction Department, Instituto Nacional de Investigación y Tecnología Agraria y Alimentaria-Consejo Superior de Investigaciones Científicas (INIA-CSIC), Madrid, Spain

**Keywords:** colostrum, cattle, management, farm, calving

## Abstract

Transfer of passive immunity (TPI) is key to achieving a good immunity status in newborn calves. The traditional scientific approach examines risk factors for the failure of TPI, but the benefits of achieving an excellent transfer of passive immunity are well recognized, justifying a closer examination of specific influencing factors. However, there is scarce information about conditions related to an excellent TPI, which may differ from those avoiding failure. Therefore, the objective of this work was to detect factors determining an excellent transfer of passive immunity. From April to July 2022, 1,041 calves from 108 European farms from six countries were studied. Colostrum quality and level of passive immunity in calves were indirectly measured with refractometry. Data of colostrum management, dam, calf and farm conditions were recorded. A categorization of poor, fair and excellent TPI were established. Mixed-effects multinomial regression modeling was implemented at animal-level, with country and herd as random factors. Median values for colostrum variables were 3 l of volume, quality of 24.4% Brix and time to administration after birth of 2 h. Only one country achieved >40% of calves in the excellent category. Mean factors affecting excellent TPI were volume and quality of the colostrum administered. In conclusion, although most farms in Europe manage and administer adequately colostrum, there are aspects to improve to achieve more than 40% of calves within the excellent category. These key factors align with those preventing failure of TPI, although this result should be taken into account with prudence based on the limitations of the study.

## 1 Introduction

An adequate transfer of passive immunity (TPI) is essential to protect calves. Good quality colostrum in dairy cattle is traditionally considered when the immunoglobulin-G concentration is above 50 mg/ml (1), which corresponds to > 22% Brix. In turn, to avoid the so-called failure of TPI (FTPI), protected calves have been defined as those with more than 10 g/l of IgG. The presence of FTPI is associated with calf health and performance problems (2) and risks factors for FTPI include farm and dam characteristics, as well as management practices related to the newborn and colostrum. In fact, the number of lactations of the dam and the time interval from calving to colostrum first-milking, colostrum quantity and colostrum quality are factors significantly associated with FTPI (1, 3). However, controversial results have been observed among studies, probably depending on region and farm. Factors such as bacterial load in colostrum, or the timing of harvesting colostrum from the dam and age of the calf at colostrum feeding were not significant in other studies (4).

Nevertheless, in these referred studies the focus is put on the risk for FTPI and factors associated. A novel categorization of passive protection has been proposed (2), describing four levels of TPI: excellent level of TPI (≥25.0 g/l of IgG; ≥9.4% Brix), good TPI (18.0–24.9 g/l; 8.9–9.3% Brix), fair TPI (10.0–17.9 g/l; 8.1–8.8% Brix), and poor TPI (<10 g/l; <8.1% Brix). An excellent TPI (ETPI) is linked with less risk of pneumonia and overall morbidity (5) or with less risk of digestive disorders (6). Therefore, our aim should be beyond avoiding FTPI and maximizing the percentage of ETPI calves (2). To achieve this objective, it is essential to detect the factors associated with ETPI, which may be different to those factors associated to avoid a failure of transfer of passive immunity. This assumption simulates that observed for health in calves, where key factors to avoid disease, are not the same issues to be taken into account to achieve an excellent level of health, including welfare. In this case aspects such as environmental enrichment, social wellbeing, etc. are also definitive (7–9). However, there is scarce scientific information on this. Thus, the objective of this observational, cross-sectional, multisite study was to identify factors associated with an excellent transfer of passive immunity in commercial cattle farms in Europe. Our hypothesis was that risk factors demonstrated for FTPI may not influence ETPI. This work provides novel insight into colostrum and neonate management, focusing on best practice calf health management, which will help to achieve an outstanding level of practice in bovine farms in Europe.

## 2 Material and methods

A prospective, observational, cross-sectional, multicentric study was conducted, gathering information from 1,041 cow-calf cases from 108 farms advised by 27 practitioners from six countries [France (*n* = 292 cases), Germany (*n* = 129), Great Britain (*n* = 231), Italy (*n* = 50), Spain (*n* = 219) and The Netherlands (*n* = 120)].

The study comprises a convenience sample collected by practitioners during regular heard health visits between April and July 2022. Farms were selected based on the condition of being supervised by a veterinarian performing yearly a control on the level of passive immunity of the newborns, sampling blood and being able to collect on average 10 newborn calves' samples/farm during the time frame of the study. Thus, no ethical approval was sought.

Blood from all the calves was sampled by the farm-veterinarian, at least 24 h after colostrum intake. The age of the sampled calves ranged between 1 and 11 d of life, as advised in previous studies on passive immunity (10). Only healthy calves from calving up to the moment of colostrum intake were sampled, discarding those with clinical signs of any disease, depression or without an adequate daily feed intake. Natural colostrum from the dams was provided by the first milking, within the first 6 h after calving, and in some farms pooled colostrum of dams calving on the same day was used. Blood from calves was sampled into sterile serum-tubes via puncture of the jugular vein. Samples were stored at room temperature for 2 h to allow clotting, and serum was separated by centrifugation at 4,500 xg for 15 min and stored in polypropylene vials at −80°C until later analysis. Colostrum and serum samples were assessed with refractometry (HR-140N, OPTIKA, S.R.L., Ponteranica, Italy). Two drops of serum/colostrum were placed on the clean platform of the refractometer. Refractometry Brix values in serum were converted into total protein values (TP) with the formula TP (g/dl) = −1.72 + 0.84 ^*^ Brix%, inferred from values previously published (2).

Information regarding the farm, management, dam and calving was recorded by the farmers and farm veterinarians at the time of calving, and then retrospectively obtained by the researchers. Not all farms provided all information required. Information linked to farm were herd size (*n* = 710); average dairy herd yield (*n* = 614); access to pasture during the dry period (*n* = 322); prepartum anionic salts administration (*n* = 308); type of calf housing (individually, collective pens or mixed system; *n* = 567) and type of bedding (on straw or hay, on soil, on sand; *n* = 446).

Information on dam/calving were parity of the dam (*n* = 139 cases); yield and length of the previous lactation (*n* = 89); dry period length, pregnancy length (*n* = 139); type of calving (not assisted, assisted and intensively-assisted; *n* = 840) and night or day birth (*n* = 633).

Data regarding the calf and colostrum management were calf breed (dairy or crossbred; *n* = 864); calf sex (*n* = 906); calf birth weight (*n* = 559); frozen or fresh colostrum (*n* = 709); type of colostrum feeding (suckled; bottle/bucket with teat; bucket without teat or esophageal tube; *n* = 875); colostrum volume (*n* = 842); time from birth to colostrum administration (*n* = 749); colostrum quality (*n* = 892) and age at blood sampling (*n* = 824).

### 2.1 Statistical analysis

The minimum amount of 225 calves with ETPI vs. 675 calves without ETPI was required based on the following assumptions: an expected herd-level prevalence of an ETPI of 30%, considering a confidence level of 95% with a precision estimate of 10% and accepting an α-risk of 0.05 and a statistical power level >0.8 in a bilateral contrast. Under these conditions we were able to detect a minimum OR of ≥1.6 for any studied factor (11).

SPSS^®^v.29 (IBM, Armonk, NY, USA) and R Statistical Softwarev.4.3.1 (R Core Team, Vienna, Austria) were used for the statistical univariate analyses. Candidate factors were tested for their association with the TPI-level, initially by using χ^2^ test/Fisher's exact test with k × 3 tables or ANOVA/Kruskal-Wallis test.

The level of the passive immunity was divided into 3 categories: (1) “poor” with calf serum Brix values <8.4%, (2) “fair” with Brix values between 8.4 and 9.4%, (3) “excellent” with >9.4%Brix, complying with previous studies for the description of excellent TPI (>9.4% Brix) (2), and for the description of failure of TPI (<8.4% Brix) (12). Both published classifications were combined by the authors proposing the categorization used in the current study, adapted to the expected results. The equivalent total protein values in serum for each category were <5.3 g/dl total proteins for the category “poor”; between 5.3 and 6.2 g/dl for the category “fair” and >6.2 g/dl total proteins for the “excellent” category.

Factors with statistical significance with *P* < 0.20 were incorporated into mixed-effects multinomial regression modeling (R mclogit package; mblogit function). Regression was implemented at animal-level, with country and herd as random factors. For random factors, the cluster variance was provided as intraclass correlation. For the model analyzing the effect of the factors on the passive immunity categories, variables changing the effect of the remaining coefficients by ≥10% (confounders) were kept in the model. The same modeling procedure was implemented to explore the effect of the same factors on the quality of colostrum administered, to explore disparities among factors effects on TPI or colostrum quality.

## 3 Results

The percentage of calves in each TPI category in total and by countries is shown in Table 1. In total, 35.3% of the calves were in the best category through a maximal transfer of maternal passive immunity. Looking at the percentage of animals in the excellent category, Spanish farms included in the study performed better, while German and French herds performed lower than the average of all calves considered.

**Table 1:** Percentage of calves in each TPI category measured on calf serum with refractometry by countries, in a multicentric study on factors associated to the transfer of an excellent passive immunity.

**Country**	**No of vets**	**Calves**	**Poor-TPI (**<**8.4% Brix;**		**Fair-TPI (8.4–9.4% Brix;**		**Excellent-TPI (**>**9.4% Brix;**	
	**and farms**	**(n)**	<**5.4 g/dl total protein**		**5.4–6.2 g/dl total protein**		>**6.2 g/dl total protein)**	
	**(n; n)**		**in calves serum)**		**in calves serum)**		**in calves serum)**	
			**Percentage**	**95% CI**	**Percentage**	**95% CI**	**Percentage**	**95% CI**
France	5; 37	292	35.6%^*^ (104/292)	30.1–41.1%	33.9% (99/292)	28.4–39.4%	30.5%^*^ (89/292)	25.2–35.8%
Germany	2; 10	129	36.7%^*^ (46/129)	27.3–44.0%	42.6% (55/129)	34.0–51.3%	21.7%^*^ (28/129)	14.5–28.9%
Great Britain	5; 27	231	23.8% (55/231)	18.3–29.3%	41.1% (95/231)	34.7–47.5%	35.1% (81/231)	28.9–41.3%
Italy	3; 13	50	14.0%^*^ (7/50)	4.0–24.0%	48.0% (24/50)	33.7–62.3%	38.0% (19/50)	27.1–51.9%
Spain	6; 9	219	15.5%^*^ (34/219)	10.7–20.4%	37.0% (81/219)	30.54–43.4%	47.5%^*^ (104/219)	40.8–54.2%
The Netherlands	6; 12	120	25.8% (31/120)	17.9–33.8%	35.8% (43/120)	27.1–44.5%	38.3% (46/120)	29.5–47.2%
Total	27; 108	1,041	26.6% (277/1,041)	4.0–44.0%	38.1% (397/041)	27.1–62.3%	35.3% (367/1,041)	14.5–54.2%

TPI, Transfer of Passive Immunity. Asterisks (^*^) indicate statistically significant difference between the marked percentage and the total percentage in the same TPI category.

Median or average values for the variables previously demonstrated as relevant regarding the ETPI were: colostrum volume administered, 3 L (Interquartile interval or IQI:2 L), colostrum quality, 24.4% Brix (IQI:6%), and average hours to first feed 3.1 (SD = 3.3 h). Calves were sampled on the fourth day of life as median value (IQI:2 d; *n* = 212 within the first 2 days of life; *n* = 38 calves with > 7 d old) to assess the level of passive immunity. The descriptive results for the average values of the assessed numerical variables and percentages of those categorical characteristics are summarized in the Supplementary Tables 1, 2. The values of the numerical variables statistically differing among countries are summarized in Table 2. Moreover, the univariate associations between factors and TPI are summarized in Table 3.

**Table 2:** Numerical variables potentially related to an excellent transfer of passive immunity statistically differing among countries, in a multicentric study on factors associated to the transfer of an excellent passive immunity.

	**France**		**Germany**		**Italy**		**The Netherlands**		**Spain**		**United Kingdom**		**Total**	
	**N**	**Average ±SD**	**N**	**Average ±SD**	**N**	**Average ±SD**	**N**	**Average ±SD**	**N**	**Average ±SD**	**N**	**Average ±SD**	**N**	**Average ±SD**
Total proteins in calf serum (g/dl)	292	5.8 ± 0.9^b^	129	5.6 ± 1.0^b^	50	6.1 ± 0.8^a^	120	5.8 ± 1.1^b^	219	6.1 ± 0.8^a^	231	6.0 ± 1.0^a^	1,041	5.9 ± 0.9
Colostrum volume (l)	183	2.8 ± 1.2^b^	113	3.0 ± 0.9^b^	75	3.3 ± 0.8^a^	119	3.1 ± 1.1^a^	202	2.8 ± 1.0^b^	150	3.1 ± 0.9^b^	842	3.0 ± 1.0
Colostrum quality (% Brix)	293	23.2 ± 5.0^b^	130	22.9 ± 4.9^b^	74	26.8 ± 4.1^a^	120	24.5 ± 3.4^a^	219	24.1 ± 4.1^a^	56	23.6 ± 3.4^b^	892	23.9 ± 4.5
Time from birth to colostrum administration (h)	142	3.4 ± 3.2^a^	117	4.1 ± 4.7^a^	75	4.5 ± 3.7^a^	120	2.2 ± 2.0^b^	209	2.3 ± 2.5^b^	86	3.5 ± 3.0^a^	749	3.1 ± 3.3
Age at calf blood sampling (days)	135	4.0 ± 1.4^a^	130	3.1 ± 1.5^b^			120	4.2 ± 1.7^a^	219	3.8 ± 2.0^a^	220	4.2 ± 1.6^a^	824	98.9 ± 42.3
Calf birth weight (kg)	132	41.8 ± 5.5^b^	108	43.9 ± 7.4^b^	70	42.8 ± 6.0^b^	118	53.6 ± 11.6^a^			131	42.1 ± 6.2^b^	559	44.9 ± 9.0
Herd size (adult cows)	113	93 ± 25^d^	90	317 ± 384^c^	70	482 ± 273^b^	120	220 ± 91^c^	219	1,401 ± 1,282^a^	98	543 ± 682^b^	710	647 ± 932
Herd Average Yield (l 305 d)	21	10,500 ± 1,123^b^	120	9,576 ± 1623^b^	75	12,270 ± 731^a^	110	9,619 ± 1,100^b^	219	12,648 ± 1,628^a^	69	8,597 ± 2,545^b^	614	10,930 ± 2,239

Superscripts indicate statistically significant difference between the figures with different letters in the same raw, with a *P*-value of < 0.001.

**Table 3:** Significance of the univariate association between factors and the level of transfer of passive immunity in newborn calves, in a multicentric study on factors associated to the transfer of an excellent passive immunity.

			***P*-value**	**df^a^**
Factors associated to farm management	Country		<0.001	10
	Practitioner		<0.001	50
	Herd size		<0.001	
	Herd yield		<0.001	
	Anionic salts addition		<0.001	2
	Pasture access at dry period		0.250	2
	Type of housing^b^		0.008	4
	Type of bedding^c^		<0.001	6
Factors associated to dam/calving	Parity	As a numerical variable	0.138	
		Categorical (binomial): Primi- vs. multiparous	0.025	2
		Categorical (three categories): Primi- vs. second- vs. multiparous	0.109	4
	Yield previous lactation		0.001	
	Lactation length		0.076	
	Pregnancy length		0.041	
	Dry period length		0.001	
	Type of calving^d^		0.156	4
	Night/day calving		0.042	2
Factors associated to calf/colostrum	Calf breed (dairy vs. beef)		0.002	2
	Calf sex		0.603	2
	Calf birth weight		0.568	
	Fresh vs. frozen colostrum		0.002	2
	Type of administration^e^		0.124	6
	Colostrum volume	As a numerical variable	<0.001	
		<2 L vs. 2–2.9 L vs. 3–3.9 L vs. >4 L vs. *ad libitum*	<0.001	8
	Colostrum quality	As numerical variable	<0.001	
		Categorical (binomial): ≤ 21 Brix% vs. >21%	<0.001	2
		Categorical (three categories): <21% vs. 21–24% vs. >24%	<0.001	4
	Time birth to colostrum administration		<0.001	
	Time birth to blood sampling		0.071	

^a^Degrees of freedom, not available for numeric variables; ^b^Type of calf housing: individual vs. collective-pairs pens vs. mixed system; ^c^Type of bedding: straw/hay vs. soil vs. sand; ^d^Type of calving: not assisted vs. assisted vs. intensively-assisted; ^e^Type of colostrum administration: suckled vs. bottle/bucket with teat vs. bucket without teat vs. esophageal tube.

According to the regression modeling results (Table 4), less factors than those resulting significant in the univariate analyses, were actually influencing an excellent level of TPI in the calves. The categories for the level of transfer of passive immunity considered were an excellent TPI (values in calf serum of >9.4% Brix), a fair TPI (with values between 8.4 and 9.4%) and a poor TPI (with values <8.4%). We observed that calves that received more colostrum were more likely to be in better TPI categories. In fact, for every liter increase in colostrum intake, the odds of having an excellent TPI instead of a poor TPI increased by 1.5 times. Similarly, for every liter increase in colostrum intake, the odds of having an excellent TPI instead of a fair TPI increased by 1.38 times. Likewise, for every Brix point higher in the colostrum quality, the odds of having an excellent TPI instead of a poor TPI increased by 1.2 times and for every Brix point higher in the colostrum quality, the odds of having an excellent TPI instead of a fair TPI increased by 1.09 times. Conversely, the later the blood was sampled after birth, the lower the probability of including the calf in the excellent category of passive immunity. For every day of delay in sampling the calf after birth, the odds of including the calf in the excellent TPI category, instead of in the poor TPI category decreased by 0.798 times. The time elapsed from birth to colostrum administration was maintained in the model, since its exclusion significantly modified the coefficients.

**Table 4:** Mixed-effects multinomial regression modeling results on factors affecting the level of passive immunity transferred to newborn calves, in a multicentric study in Europe.

				**95% CI**	
	**β**	***P-*value**	**Exp (β)**	**Lower**	**Higher**
**Equation 1: Poor vs. Excellent**					
**Poor-TPI:**<**8.4% Brix;**<**5.3 g/dl TP; Excellent-TPI:** >**9.4% Brix;** >**6.2 g/dl TP**					
Colostrum intake (l)	0.440	<0.001	1.553	1.154	2.090
Colostrum quality (%Brix)	0.182	<0.001	1.199	1.115	1.289
Days from calving to sampling	−0.224	0.005	0.798	0.681	0.936
Hours from calving to feeding	−0.012	0.772	0.987	0.905	1.076
**Equation 2: Fair vs. Excellent**					
**Fair-TPI: 8.4–9.4%Brix; 5.4–6.2 g/dl TP; Excellent-TPI:** >**9.4%Brix;** >**6.2 g/dl TP**					
Colostrum quality (%Brix)	0.089	0.009	1.094	1.022	1.171
Days from calving to sampling	−0.110	0.111	0.895	0.781	1.026
Hours from calving to feeding	−0.006	0.867	0.993	0.922	1.070
**Equation 3: Poor vs. Fair**.					
**Poor-TPI:**<**8.4% Brix;**<**5.3 g/dl TP; Fair-TPI: 8.4–9.4% Brix; 5.4–6.2 g/dl TP**					
Colostrum intake (l)	0.138	0.298	1.146	0.885	1.484
Colostrum quality (%Brix)	0.092	0.002	1.097	1.032	1.165
Days from calving to sampling	−0.100	0.158	0.904	0.786	1.039
Hours from birth to administration	−0.001	0.966	0.998	0.928	1.073
	**Variance (SD) equation 1**	**Variance (SD) equation 2**		**Variance (SD) equation 3**	
Country	0.057 (0.001)	0.113 (<0.001)		0.103 (0.001)	
Country: herd	0.007 (<0.001)	0.035 (<0.001)		0.025 (<0.001)	

TPI, Transfer of Passive Immunity; TP, calves serum Total Protein. Random-effects cluster variance provided with standard deviation (SD). β, regression coefficient; Exp (β), regression coefficient exponential (=Odds Ratio).

Some studied factors were not significant in the model for TPI but affected colostrum quality (Supplementary Table 3). These were access to pasture during the dry period and intensively-assisted calving, which were associated with poorer colostrum quality (*P* = 0.012; OR = 0.283; CI 95%: 0.105–0.761, and *P* = 0.042; OR = 0.492; CI 95%: 0.98–0.244, respectively). In fact, the access to pasture by the dam during the dry period was linked to a decreased odds to produce a colostrum of excellent quality (>24% Brix value) by 0.283 times. Similarly, a dystocic calving was associated with a decreased quality of colostrum by 0.492 times, compared to a normal calving. All other factors did not affect TPI.

## 4 Discussion

Our results associate colostrum quantity and quality to an excellent level of passive immunity in the studied European farms under the described conditions, while intensively-assisted calving and access to pasture during the dry period were associated with a decreased in colostrum quality.

We observed differences by country regarding how many calves achieved an excellent TPI. The percentage of ETPI calves was 35.3% in average, like that previously reported in one single German farm (5). The consensus level of the percentage of calves with an excellent passive immunity in dairy farms is >40% to minimize the morbidity (2). Only one country in our study achieved with this cut-off value. Therefore, we can conclude that enhancing the percentage of ETPI calves is a challenge on European farms, and more attention should be paid to this.

Based on the convenience selection of farms, our data is not representative of all farms in each region, however several farms per country from different regions and a notable number of calves per farm were included, such that the study gives a valuable description of each country. Limitations of this study are the convenience selection of the farms and the uneven number of calves and farms per country. Based on this, further studies are needed including more calves and farms per country in a observational study on randomly selected farms and calves. Nevertheless, we consider that it is still possible to make inference about the management of the farms selected, with an average of 10 calves per farm sampled. Regarding our statistical analyses, testing a large number of variables may have introduced a risk of having a type 1 error or false-positive results. Although, the statistical model corrected this error in part, there is no way to assure that colostrum management practices can be considered independent, and therefore, that the factors studied were independent. Hence, the conclusions should be taken with prudence.

Above 25 g/l serum IgG (2) are required, which means the administration of at least 200 g IgG (13) in the first 24 h after birth, which logically depends on the colostrum volume and quality, but also on the weight of calves. In our study, ETPI calves received a median of 3 l of colostrum (IQI:1.13 L) with 24.4% Brix (IQI:4.5%). It means a calculated average of 73.5 g/l IgG and 228 g IgG administered in the first 2.5 h after birth, which already sufficiently addresses the previously discussed minimum values for the first 24 h (13). The fact that in our study most of the farms provided high quality colostrum (>50 g/l IgG; >22% Brix), as described by Lichtmannsperger et al. (1), may explain that the main contributory factor to an ETPI was the volume. The study was performed at commercial farms, without altering their usual management, and reflects that some calves receive a scarce volume of colostrum, routinely, in these farms. This evidence generates an easy recommendation to enhance the percentage of ETPI calves. However, we should keep in mind that providing high quality colostrum to all calves may be challenging, especially in primiparous cows (14, 15). Therefore, we must reiterate the point that ensuring high quality of colostrum is highly important to achieve an excellent level of TPI.

In our study two factors decreased colostrum quality: being on pasture during the dry period, probably reflecting problem of an imbalanced nutrition (16), and the level of calving assistance, similarly to that previously observed (17). In contrast to our results, no difference was found in the colostrum quality determined with the content of immunoglobulins, of investigated cows after suffering dystocia (18, 19). Our results may be related to a poor condition of the dam previous to calving, fact that can be associated to dystocia and indirectly to a poorer colostrum quality, once the mean risk factors such as calf birthweight and maternal pelvic size (20) are controlled. An alternative hypothesis could be the decreased welfare demonstrated in the cows having dystocia pre and post-calving (21), which may have affected the production of an adequate quantity and quality of colostrum. However, these are speculative considerations on the results and more research should be conducted.

Although we have demonstrated this negative effect of assisted calvings on the colostrum quality through the model analyses, it is of note that we observed the opposite in the univariate analyses on the TPI in calves, with assisted calvings showing 10 percentage-points more ETPI calves than the average (data not shown), which is contrary to that observed by other researchers (22). This result in our data may be explained by the fact that assisted calving results in a less delayed administration of colostrum. In fact, calves born after assisted calving in our study were fed colostrum 1 h after birth (IQI:1.5 h) while those born without assistance were fed later (2.5 h; IQI:3 h). Even though, dystocia seems to influence negatively the level of passive immunity of the calves (23) due to the respiratory acidosis (24) or to a decreased immunoglobulins absorption (22). By contrast, in a former research no impaired absorption of immunoglobulins by dystocic delivered calves was observed and it was hypothesized with the possible effect of the endogenous cortisol seen in these calves born to assisted calvings hindering the transfer of the passive immunity (18). Therefore, further studies are needed.

The transfer of passive immunity may be affected by other factors besides colostrum quantity and quality (1). Our univariate analyses detected many factors as significant contributors to TPI, however, once all circumstances were analyzed together, only colostrum quantity and quality remained significant. Although the regression model prevents the inclusion of redundant variables, it is not possible to assure the absolute independence of all the aspects implicit in the colostrum management. Therefore, as stated previously, our results need to be considered with prudence. The time from birth to colostrum administration has been described as an important risk factor for FTPI in aforementioned studies. In our research, median time was 2 h (IQI:3 h), which is an adequate interval for almost all the calves studied. Moreover, some calves with a lower level of passive immunity had ingested colostrum after a short interval of time after birth, which supports that this factor did not relate to the TPI, at least not under the circumstances of our study. This does not mean that time to colostrum administration is not relevant to avoid a failure of passive immunity, as previously demonstrated (1), but that, under the described conditions, it was not relevant to achieve an excellent level of passive immunity by the calves. Moreover, type of colostrum feeding did not affect TPI, with colostrum volume having been included in the analysis model and no effect was observed by colostrum fed via esophageal tube, in contrast to earlier findings (25).

Based on our results, we can conclude that farms in European countries on average manage and administer colostrum adequately to calves. However, certain practices should be improved to achieve the goal of >40% of calves with excellent passive immunity. We explored the possibility of finding factors determining an excellent level of passive immunity in calves, that may be different from those relevant for a poor transfer of passive immunity. Conversely to our hypothesis, we could only detect significant factors associated with colostrum quantity and quality for an excellent level of passive immunity, which are the same key factors identified to avoid a poor TPI. Although our study requires consideration of certain limitations when assessing conclusions, the approach focussing on an excellent level of passive immunity in the European farms is novel and of value for the cattle sector.

## Data Availability

The raw data supporting the conclusions of this article will be made available by the authors, without undue reservation.
