# Supplementary material for: Factors associated with an excellent transfer of passive immunity: multisite, cross-sectional study conducted in different European countries on dairy cattle
Source: Front Vet Sci. 2025 Feb 25;12:1515196. doi: 10.3389/fvets.2025.1515196 (PMC11894572; doi:10.3389/fvets.2025.1515196)
Supplement: Supplementary file 1 [file Table_1.docx]

**Supplementary Table 1: Descriptive data of newborn calves and studied, numerical factors possibly associated to the efficiency of the transfer of passive immunity, in a multicentric study in Europe.**

| **Factors associated to farm** | N | Mean |  | SD | Median | Min | Max |
| --- | --- | --- | --- | --- | --- | --- | --- |
| Herd size (adult cows, farms with data) | 108 | 647 | ± | 932 | 257 | 45 | 3670 |
| Herd average milk yield (l, 305d, farms with data) | 8 | 10930 | ± | 2239 | 10788 | 3500 | 15015 |
| **Factors associated to dam/calving** |  |  |  |  |  |  |  |
| Parity of the dam (number) | 129 | 2.40 | ± | 1.39 | 2 | 1 | 6 |
| Yield previous lactation (kg) | 89 | 13400 | ± | 3569 | 13223 | 6637 | 22113 |
| Length previous lactation (days) | 89 | 355 | ± | 62 | 347 | 217 | 547 |
| Dry period length (days) | 89 | 63.83 | ± | 24.45 | 60.00 | 22.00 | 166.00 |
| Pregnancy length (days) | 139 | 279 | ± | 6 | 280 | 246 | 292 |
| **Factors associated to calf/colostrum** |  |  |  |  |  |  |  |
| Calf birth weight (kg) | 559 | 44.87 | ± | 8.96 | 44 | 28 | 90 |
| Colostrum volume (l) | 842 | 2.98 | ± | 1.03 | 3.00 | 0.50 | 7.50 |
| Colostrum quality (%Brix) | 892 | 23.87 | ± | 4.52 | 0.15 | 10.10 | 40.00 |
| Time from birth to colostrum administration (h) | 749 | 3.12 | ± | 3.29 | 0.12 | 2.00 | 23.00 |
| Total proteins in calf serum (g/dl) | 1041 | 5.90 | ± | 0.94 | 5.84 | 2.56 | 9.20 |
| Age at calf blood sampling (days) | 824 | 98.93 | ± | 42.29 | 1.47 | 1.00 | 11.00 |

**Supplementary Table 2: Descriptive data of newborn calves and categorical risk factors possibly associated to the efficiency of the transfer of passive immunity, in a multicentric study in Europe.**

| **Factors associated to farm management** | **N** | **Category, percentage (n/N)** | | |
| --- | --- | --- | --- | --- |
| Access to pasture during the dry period | (26 farms; 322 calves) | Yes: 22.7% (73/322) | No: 77.3% (249/322) |  |
| Prepartum anionic salts administration | (25 farms, 308 calves) | Yes: 51.3% (158/308) | No: 48.7% (150/308) |  |
| Type of calf housing (individually, collective pens or mixed system) | (46 farms; 567 calves) | Individually: 82.2% (471/573) | Pens: 10.1% (58/573) | Mixed: 7.7% (44/573) |
| Type of bedding (on straw or hay, on soil, on sand) | (36 farms; 446 calves) | Straw/hay: 85.8% (388/452) | Soil: 11.1% (50/452) | Sand: 3.1% (14/452) |
| **Factors associated to dam/calving** |  |  | | |
| Type of calving (not assisted, assisted and intensively-assisted by a veterinarian) | 840 calves | Not Assisted: 75.6% (664/878) | Assisted: 19% (167/878) | Vet Assisted: 5.4% (47/878) |
| Night or day birth | 633 calves | Night: 28.4% (194/683) | Day: 71.6% (489/683) |  |
| **Factors associated to calf/colostrum** |  |  | | |
| Calf breed (dairy or crossbred) | 864 calves | Dairy: 69.9% (604/864) |  | Crossbred: 30.1% (260/864) |
| Calf sex (n=906); | 906 calves | Female: 58.1% (551/948) |  | Male: 41.9% (397/948) |
| Frozen or fresh colostrum (n=709); t | 709 calves | Frozen: 13.8% (100/723) |  | Fresh: 86.2% (623/723) |
| Type of colostrum feeding (suckled from the dam; bottle/bucket with teat; bucket without teat; oesophageal tube) | 875 calves | Bottle/Bucket: 47.1% (419/889) | Suckled: 24.4% (217/889) |  |
|  |  | No teat: 4.5% (40/889) | Tube: 24% (213/889) |  |

**Supplementary Table 3: Mixed-effects multinomial regression modelling results on risk factors for the colostrum quality in a study on transfer of passive immunity in calves, in a multicentric study in Europe.**

| Equation 1: “<21%” vs. “21-24%”  <21% for bad quality colostrum, 21-24% for adequate quality colostrum. | | | | | | | |
| --- | --- | --- | --- | --- | --- | --- | --- |
|  |  |  |  | | 95 % CI | | |
|  | β | *P*-value | Exp (β) | | Lower | Higher | |
| Type of calving (hard/veterinary assisted)* | -0.440 | 0.730 | 0.644 | | 0.052 | 7.955 | |
| Type of calving (assisted)* | -0.127 | 0.795 | 0.881 | | 0.336 | 2.308 | |
| Pasture (yes)** | -1.126 | 0.052 | 0.324 | | 0.082 | 1.045 | |
| Equation 2: “<21%” vs. “>24%”;  <21% for bad quality colostrum, >24% for excellent quality colostrum. | | | | | | | |
|  |  |  |  | | 95 % CI | | |
|  | β | *P*-value | Exp (β) | | Lower | Higher | |
| Type of calving (hard/veterinary assisted)* | -0.709 | 0.042 | 0.492 | | 0.244 | 0.980 | |
| Type of calving (assisted)* | -0.030 | 0.947 | 0.969 | | 0.394 | 2.387 | |
| Pasture (yes)** | -1.262 | 0.012 | 0.283 | | 0.105 | 0.761 | |
|  | Variance (SD) equation 1 | | | Variance (SD) equation 2 | | |  |
| Country | 0.409 (<0.001) | | | 0.230 (<0.001) | | |  |
| Country:herd | 0.004 (<0.001) | | | 0.003(<0.001) | | |  |

*Not assisted is the base. ** No pasture is the base
